# Bioremediation potential of *Pseudomonas fluorescens* strain isolated from the Ha'il region of Saudi Arabia

**DOI:** 10.6026/97320630019901

**Published:** 2023-09-30

**Authors:** Abdulmohsen K. D. Alsukaibi, Lassaad Mechi, Fathi Rabeh Alimi, Asma K. A. A. Alshamari, Eida M. Alshammari, Otaibi Ahmed A., Subuhi Sherwani, Mohd Wajid Ali Khan

**Affiliations:** 1 Department of Chemistry, College of Sciences, University of Ha'il, Ha'il 55473, Saudi Arabia; 2 Medical and Diagnostic Research Center, University of Ha'il, Ha'il, 55473, Saudi Arabia; 3 Department of Biology, College of Sciences, University of Ha'il, Ha'il 55473, Saudi Arabia

**Keywords:** *Pseudomonas fluorescens*, Ha'il, Detoxification, Bioremediation, Water pollutants, Toxicants, Heavy metals, pesticides

## Abstract

Increased amounts of toxicants may cause sever health issues in humans as well as in aquatic life. Scientists are developing new technologies to combat these problems. Biological methods of detoxification are always beneficial for the environment. Pseudomonas fluorescens is known for its detoxification capacity. In this study *Pseudomonas fluorescens* stains were isolated from different locations of the Ha'il region, Saudia Arabia. The microbial strain AM-1 displayed resistance to heavy metals (Cr6+, Ni2+, Cd2+, Pb2+) and pesticides (BHC, 2,4-D, Mancozeb) at pollutant levels typical of highly contaminated areas. Additionally, AM-1 exhibited substantial detoxification potential, reducing toxicity by 40.67% for heavy metals and 47.4% for pesticides at 3x concentrations. These findings suggest that the AM-1 strain supports environmental remediation and pollution mitigation. Atomic absorption spectrometry (AAS) results exhibited bioremediation efficiency for metals Cr^6+^, Ni^2+^, and Pb^2+^ using immobilized cells of *P. fluorescens* AM-1 isolate, estimated to be 60.57%, 68.4%, and 53.93% respectively. These findings show that AM-1 strain has a potential role in bioremediation of water pollutants and may have future implications in wastewater treatment.

## Background:

The confluence of technological innovation, rapid urbanization, evolving consumption patterns, burgeoning population growth, and rapid socioeconomic development has unequivocally engendered heightened levels of water pollution within the biosphere, thereby exacerbating environmental degradation [1]. The main culprits responsible for this escalating environmental concern are the effluents stemming from domestic and industrial activities, constituting the principal contributors to the natural burden of water pollution, replete with heavy metals, dyes, and pesticides [2]. This burgeoning pollution burden poses a challenge in the realm of wastewater management, yielding not only a substantial escalation in treatment costs but also the introduction of a diverse array of chemical contaminants into our precious water sources [3]. *Pseudomonas fluorescens* is generally a harmless bacterium that inhabits various environments like soil, water, and plant surfaces [4]. This bacterium has unique physical and genetic characteristics that make it a valuable resource in fields like biotechnology, agriculture, and environmental clean-up [5-6]. Bioremediation, the process of cleaning up toxic substances that could harm public health, is facilitated by microbial communities [7]. Dangerous substances like heavy metals, pesticides, and phenols are significantly hazardous to living things, as may interfere with cellular functions by reacting with proteins, nucleic acids, and phospholipids [2]. Heavy metals are everywhere and are long-lasting pollutants in the environment, which come from human activities and the main sources are oil refineries, petrochemical plants, chemical industry, and mining [9]. Pseudomonas comprises about 200 species [10] and some of them showed the capacity to endure large quantities of heavy metals, measured in millimoles [11-12]. Pesticides can accumulate in crop remains, municipal waste, farm manure, and soil due to their use in industry, military operations, homes, and agriculture [13]. The application of insecticides and herbicides in farming can also result in the buildup of pesticides in soil, which may break down slowly. Indeed, certain pesticides, including DDT, BHC, heptachlor, and mancozeb, are known for their high stability, allowing them to persist in the environment for extended durations [13]. This stability raises concerns about their potential long-term environmental impact and the need for effective strategies to mitigate their presence and effects. They can also accumulate fatty tissues when food contaminated with these residues is consumed. Additionally, these toxic pesticides and can cause several negative effects, such as immunological disorders, damage to the liver, kidney, thyroid, and lungs, and can be associated with porphyria [14,15]. Therefore, it is of interest to focus on the bioremediation and removal of some major pollutants through the use of a strain of *Pseudomonas fluorescens* that was isolated from a waste dump site of Ha'il region.

## Materials and Methods:

## Chemicals:

Peptones, tryptone, agar-agar, magnesium sulphate (MgSO_4_), dipotassium hydrogen phosphate (K_2_HPO_4_), Copper sulphate (CuSO_4_), nickel sulphate (NiSO_4_). glacial acetic acid, manganese ethylene bis dithiocarbamate (mancozeb), benzene hexachloride (BHC) and 2,4-dichlorophenoxy acetic acid (2,4-D), Cadmium chloride (CdCl), magnesium sulphate (MgSO4), calcium chloride (CaCl_2_), lead acetate (Pb(C_2_H_3_O_2_)_2_), potassium chromate (K2HPO4) were purchased from Sigma Aldrich USA.

## Media:

The pure culture of *Pseudomonas fluorescens* was grown in a medium with mineral salt that was dissolved in sterile distilled water. Gradients used in the medium are peptone (10 gm), tryptone (10 gm), magnesium sulphate (MgSO_4_) (1.5 g), K2HPO_4_ (1.5 g), and glycerol (10 mL) and then make up the volume to 1 L. Nutrient broth (7 g/l) used to prepare plates for counting colonies.

## Extracting *Pseudomonas fluorescens* from soil contaminated with pollutants:

Samples of soils were obtained from waste dump sites in Ha'il region, Saudi Arabia. Initially, 25 grams of the soil sample was mixed with 100 milliliters of water (distilled), then shaken and left for settling. Supernatants were collected and pellets were discarded. The enrichment method was used for the isolation of desired bacterial strain from soil samples. A *Pseudomonas fluorescens* medium was prepared using 10 g of tryptone, 1.5 g of MgSO_4_, 1.5 g of K2HPO_4_, and 10 ml of glycerol was added to distilled water and autoclaved at 121°C for 30 mins. After cooling of the media, 100 µl of soil sample supernatants were added to each flask and left overnight at 37°C in the shaking incubator. Next day, nutrient broth was used to prepare agar plates and these were streak with 20 µl samples from different cultures. Cultures with toxicants were used to isolate the resistant bacteria. Eventually, a single clone of *Pseudomonas fluorescens* strain (AM-1) was isolated and was chosen for more experiments.

## Preparation of different concentrations of toxicants:

The objective of this study was to address the issue of water pollution caused by heavy metals and pesticides and bioremediation enabling detoxification of these pollutants. To achieve this, various experiments were conducted with these pollutants alone or in combinations. The maximum concentrations of these individual pollutants were selected based on previous report [16]. The study considered the concentration of various heavy metals as 1x, where Cd^2+^ was 12 ppm, Cr^6+^ was 10 ppm, Ni^2+^ was 300 ppm, and Pb^2+^ was 194 ppm. The study also used double and triple concentrations of these metals, which were referred to as 2x and 3x, respectively. The mixture of all these heavy metal toxicants was mixed in a concentration of 1x, 2x, and 3x. Toxicants concentrations were prepared using the following formulas.

PPM calculations- mass of solute/mass of solution x 106

PPB calculations- mass of solute/mass of solution x 109

The experiment involved taking pesticides (1x), which were selected based on their concentrations in regions where they are heavily present [16]. Specifically, 78 ppb of 2,4-D, 500 parts per billion (ppb) of BHC, and 312 ppm of mancozeb were used. Similarly, 200% (2x) and 300% (3x) concentrations of these pesticides were also prepared accordingly.

## Measurement of the isolate's growth rate in response to stress:

Stressful conditions were developed to observe the growth curve of the resistant bacterial strain. In the experiment, liquid Pseudomonas broth (10 ml) was distributed into glass flasks. To these flasks, pure bacterial culture (0.3 ml) then introduced heavy metals with various concentrations (1x, 2x, and 3x) and similarly for pesticides. Bacteria were also grown in another condition where both toxicants (heavy metals and pesticides) were mixed in varying concentrations (1x, 2x, and 3x). The flasks were then kept at a temperature of 37°C, and the turbidity change was recorded at 540 nm after specific time intervals of 4, 8, 12, 24, and 48 hours. To serve as a baseline or negative control, pure culture without any exposure to toxicants was also included in the study. This experimental design allows for the assessment of how these substances and their combinations affect the microbial culture's growth and metabolic activity over time.

## Survival rate of *Pseudomonas fluorescens* strain under stressful conditions:

In this experiment, an overnight culture (0.2 ml) of *Pseudomonas fluorescens* was transferred into a broth (10 ml) and leave for the culture to grow on the optimum temperature (37°C) until the optical density (O.D.) reached twice read at 540 nm. Then using centrifugation (5000rpm x 5 mins) bacterial cells were collected. Supernatant was discarded and to the pellet 10 ml normal saline was added. Various testing concentrations of toxicants were added. These cells were then incubated at 37°C for 6 hours. Subsequently, serial dilutions (100 folds) of the samples were done. From these dilutions, a 0.1 mL aliquot was spread onto agar plates. These plates were then incubated overnight at 37°C to allow for colony formation. Finally, the number of bacterial colonies that developed on the plates was counted. This experimental approach is used to assess the impact of different concentrations of toxic substances on bacterial growth and survival.

## Immobilization of the test sample within Alginate gel spheres:

The Khan and Ahmad [16] method was employed to immobilize cells using alginate. For the immobilization process, 0.1 mL of a cell suspension was blended with 0.9 mL of a 2% sodium alginate solution at 25°C. This mixture was then dispensed from a syringe into a 250 mL solution of calcium chloride (0.8 M). The resulting beads were extracted, washed, and subsequently exposed to various concentrations of toxic substances overnight at room temperature. Finally, the beads were taken out from the toxicant solutions, and solutions were examined to assess the extent of bioremediation or detoxification achieved by the immobilized cells.

## *Allium cepa* test:

The Fiskesjo method [17] was used to carry out an experiment on the model water, which involved testing different concentrations of toxicants using *Allium cepa*, or small red onions of the same size. Onions were prepared by removing outer scales and keeping the primordial ring. Test tubes contained solutions of heavy metal salts and pesticides, both pre- and post-immobilization with AW1 cells, with Aqua guard water as a control. Each tube held one onion with its root in contact with the liquid. Fresh samples were added every 12 hours to ensure contact, enabling evaluation of pollutant interaction and AW1 cell system effectiveness. The onions were treated for 5 days in the dark, and their roots were measured for each concentration, with the average length of 10 roots used as the measurement. The reduction in the root elongation of *Allium cepa* served as an indicative marker of toxicity.

## Examination of high-density metals using atomic absorption spectrometry:

The concentration of heavy metals was measured both before and after treating it with the immobilized cells, using the atomic absorption spectrometer (AA-7800, Shimadzu, Japan). Standards used in the analysis were sourced from Sigma Aldrich USA. All samples were prepared with double distilled water.

## Results and Discussion:

An important and difficult problem for any growing and developing city is environmental pollution with high concentrations of heavy metals, pesticides, and several other toxicants causing serious issues. The city Ha'il is a capital of the Ha'il province and is one of the fastest growing cities in the northern region of Saudi Arabia. It has a reasonably large industrial area where numerous small industries, workshops for car and heavy vehicle repair, battery repair and replacement for light and heavy vehicles that may produce heavy metals. Because of favorable climatic condition of Ha'il region, it is an important place for agriculture too, and it is possible the presence of insecticides and pesticides in these sites. Thus, soil samples were collected from different waste dump sites of Ha'il city to isolate resistant *P. fluorescens* strains.

Heavy metals and pesticides are present in the sites and may be discharged into groundwater and arable land. Use of chemical means to detoxify these toxicants further introduces a wide range of chemical pollutants, which may be toxic to humans, aquatic life as well as to the microbes important to the ecosystem. Thus, is an urgent need to create a method for bioremediation of these toxins that can make the environment safe and help in cleaning water, making it safe for consumption? An initial step in that approach is represented by this study.

**Figure 1:**
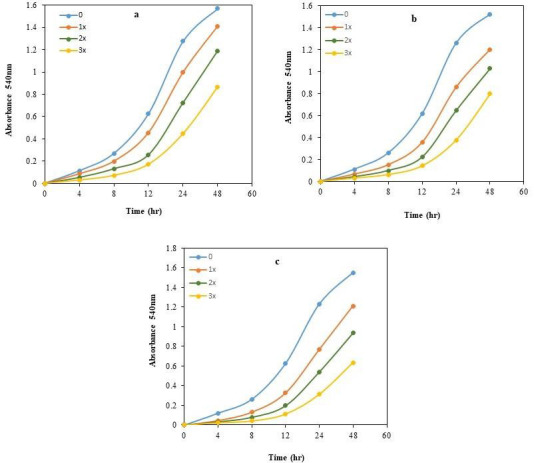
Growth curves of the *P. fluorescens* isolate in presence of heavy metals (a), pesticides (b) and combinations of both heavy metals and pesticides (c). The concentrations of the tested toxicants were as follows 1x, 2x and 3x. *P. fluorescens* without toxicant (0) serves as controls. All the values are the Mean of three independent readings

*Pseudomonas fluorescens* is a type of harmless bacteria found in soil that has the ability to biologically transform foreign substances [18]. This particular strain of bacteria can be isolated from a population of microorganisms found in soil heavily contaminated with pollutants. It is a good option for bioremediation since it looks to be highly resilient and efficient in removing heavy metals and pesticides that are frequently present in wastewater systems [12].

(Figure 1) depicts the growth pattern of Pseudomonas fluorescence in response to the co-presence of multiple heavy metals (Cd2+, Cr6+, Ni2+, and Pb2+) and pesticides (BHC, 2,4-D, and mancozeb), and combination of both in different concentrations (1x, 2x, and 3x). After a varying period of time in a dormant phase, the bacterial strain appeared to resume growth at a rate similar to that seen in the absence of toxicants. This indicates that the bacteria had developed a high level of resistance to the pollutants after a dose-dependent preparatory phase. However, the delay in growth observed in the presence of the higher concentrations of the heavy metals (Figure 1a). A similar pattern was observed in the presence of pesticides (Figure 1b). Combinations of both heavy metals and pesticides were also tested in varying concentrations (Figure 1c). Even the 3x concentrations of the combination of heavy metals and pesticides showed growth in the *P. fluorescens*, however, there was a reasonable decrease in growth compared to the growth in presence of heavy metals or pesticides alone with 3x concentrations. This could be due to excessive toxic effects of the combination of the toxicants on the bacterial cells. Bacterial growths without toxicants (0) were considered as positive control.

A second experiment was carried out to ascertain whether there was any major irreversible harm to the bacterial cells (Table 1). *P. fluorescens* cells viability was tested using the procedure provided in the materials and method section. Harvested cells from the overnight culture were suspended in sterile normal saline with or without toxicants and incubated for 6 hours at 37°C. Using serial diluted samples from these plates were prepared and left for overnight the colonies were counted. No significant change or decrease in the colonies count was observed even after increasing concentrations.

Toxicity bioassays are required to assess contaminants' negative effects. Therefore, regardless of whether organic toxicants had been biodegraded, our main target was to ascertain to decrease toxicity of polluted water samples treated with an immobilized AM-1 strain. This was significant because, according to earlier findings for instance, the P. ostreatus strain reduced the phenolic content of green olive wastewater, but it had no influence on phytotoxicity [19,20]. In this study *Allium cepa* test was employed, which is a technique for evaluating chemical contaminants that present environmental concerns [21,22]. As a result, we made the decision to use the accepted *Allium cepa* test, a method for assessing chemical pollutants that raise environmental issues. A calcium alginate gel matrix was used to contain the *P. fluorescens* AM-1 strain since prior research indicated that an immobilized system offers greater benefits than a free cell system. Using a model aqueous solution comprising typical heavy metals and pesticides, either in isolation or in tandem., a test was carried out to determine this strain's capacity to detoxify using the *Allium cepa* test. The model water's *Allium cepa* root inhibition assay findings for both before and after it was exposed to calcium alginate beads containing the *Pseudomonas fluorescens* AM-1 strain are shown in Table 2,Table 3,Table 4. According to the findings, the AM-1 immobilized cell system exhibited a maximum heavy metal detoxification effectiveness of 67.95% and a minimum of 40.67%.

The findings of the immobilized AM1 cell system treatment and the *Allium cepa* test for the toxicity of model water containing test pesticides are shown in Table 3. The immobilized cell system's maximum and minimum pesticide detoxification efficiencies were found to be 70.82% at 1x concentration which was decreased significantly at 3x concentration (47.40%).

(Table 4) presents data on the extent of detoxification achieved through the combination of toxicants. At 1x, 2x, and 3x toxicant concentrations, the observed detoxification efficiencies for combined toxicants were 56.3%, 46.02%, and 39.18%, respectively.

The removal of heavy metals from water through the utilization of the immobilized *Pseudomonas fluorescens* system within a short treatment period of 24 hours is shown in Table 5 as the results of an AAS study. The elimination of 60.57% of chromium V1, 68.4% of nickel, and 61.0% of lead exhibited strain's great potential for bioremediation.

A trustworthy method to assess the toxicity of a water system is the *Allium cepa* test [23]. The immobilized cell method was discovered to be extremely successful in detoxifying important water pollutants, as shown in Table 2,Table 3,Table 4, with remarkably high detoxification efficiencies attained in just 24 hours. The immobilized cell system specifically achieved a detoxification efficiency of up to 40.67% for heavy metals, 47.40% for pesticides, and 33.84% for a combination of all heavy metals and pesticides at concentrations twice (3x) what is expected to be present in highly polluted regions. Our research found that the *Pseudomonas fluorescens* AM1 isolate was able to remove not only cationic heavy metal species such as Cd2+ or Pb2+, but also the chromate, an extremely poisonous anionic form of hexavalent chromium (Table 5). Hexavalent chromium is known to be extremely hazardous [24], and its bioremediation requires different mechanisms than those for cationic metal species, a biosorption mechanism, for instance [25]. Hexavalent chromium must be converted to trivalent species in the case of chromate through the internalization of CrO_4_^2+^ via SO_4_^2+^ influx machinery or other mechanisms [24]. Our observations thus imply that the test isolate has at least two metal bioremediation mechanisms. Similar to this, numerous enzymatic systems would be necessary for the detoxification of organic contaminants including BHC, 2,4-D, and mancozeb. A time lag when changing from a favorable to an unfavourable environment may happen due to the intricacy of resistance mechanisms, suggesting the inducible nature of particular enzyme systems. Given the current level of water pollution especially in Asian countries [27-28,29, 30], it is imperative to create a clear, effective, and doable strategy to combat environmental degradation. Our research serves as an initial effort to eliminate pollutants from water system using a commonly found, generally non-pathogenic microorganism that grows quickly and effectively.

## Conclusion:

Data shows that the *Pseudomonas fluorescens* isolate AM-1 is resistant to heavy metals, pesticides, as well as their mixtures. The fact that *Pseudomonas fluorescens* AM-1 strain can be immobilized in alginate beads which increased the efficacy of bioremediation of heavy metals and pesticides is a noteworthy finding. However, additional investigations including a wide range of toxicants are needed to analyze bioremediation ability of AM-1 strain. As well as mechanisms involved in bioremediation processes also need to be elucidated.

## Figures and Tables

**Table 1:** *P. fluorescens*isolate viability at various toxicants concentration

**Toxicants concentrations**	**Colonies/ml**
Positive control	4.01 ± 0.21 x 10^8^
1x (heavy metals+Pesticides)	3.77±0.24x10^8^
2x (heavy metals+Pesticides)	3.57±0.22x10^8^
3x (heavy metals+Pesticides)	3.12±0.24x10^8^
Each sample was tested in triplicates. Values are given in mean ± SD. Heavy metals included in this assay are Ni2+, Cd2+, Cr6+, and Pb2+ and pesticides are mancozeb, BHC, and 2,4-D.

**Table 2:** Evaluation of the bioremediation efficacy of *P. isolates* immobilized on the alginate beads against different concentrations of heavy metals.

**Conditions**	**1X**		**2X**		**3X**		**Negative Control**
	**Length (cm)**	**Inhibition (%)**	**Length (cm)**	**Inhibition (%)**	**Length (cm)**	**Inhibition (%)**	**Length (cm)**
Model water without any treatment containing heavy metals (+Control)	0.96±0.05	86.85	0.56±0.02	92.32	0.04±0.01	99.44	7.3±0.6
Immobilized P. isolate treated model water containing heavy metals	5.92±0.12	18.9	4.88±0.13	33.15	3.01±0.08	58.77	7.3±0.36
Bioremediation efficacy	67.95%		59.17%		40.67%		-
Each value is given in mean ± SD. Each sample runs in triplicate. Each length (cm) given is average length. 1x heavy metals contain Cd^2+^ (12 ppm), Cr^6+^(10 ppm), Ni^2+^ (300 ppm), and Pb^2+^ (194 ppm). 2x and 3x of the heavy metal mean two times and three times the concentration of metals of given above. Experimental conditions without toxicants serve as negative control. Bioremediation efficacy calculation: Inhibition % in untreated water - inhibition % in immobilized *P. isolates* treated water at different heavy metal concentrations.

**Table 3:** Evaluation of the bioremediation efficacy of *P. isolates* immobilized on the alginate beads against different concentrations of pesticides.

**Conditions**	**1X**		**2X**		**3X**		**Negative Control**
	**Length (cm)**	% Inhibition	**Length (cm)**	**% Inhibition**	**Length (cm)**	**% Inhibition**	**Length (cm)**
Model water without any treatment containing pesticides (+Control)	1.26±0.07	82.74	0.76±0.03	89.59	No growth	100	7.3±0.36
Immobilized P. isolate treated model water containing pesticides	6.43±0.14	11.92	5.17±0.11	29.18	3.46±0.14	52.6	7.3±0.36
Bioremediation efficacy	70.82%		60.41%		47.40%		-
Each value is given in mean ± SD. Each sample runs in triplicate. Each length (cm) given is average length. 1x pesticides contain BHC (500 ppb), 2,4-D (78 ppb), and mancozed (312 ppm). 2x and 3x of the pesticides mean two times and three times the concentration of pesticides given above. Experimental conditions without toxicants serve as negative control.Bioremediation efficacy calculation: Inhibition % in untreated water - inhibition % in immobilized *P. isolates* treated water at different pesticides concentrations.

**Table 4:** Evaluation of the bioremediation efficacy of *P. isolates* immobilized on the alginate beads against different concentrations of combination of heavy metals and pesticides.

**Conditions**	**1X**		**2X**		**3X**		**Negative Control**
	**Length (cm)**	**% Inhibition**	**Length (cm)**	**% Inhibition**	**Length (cm)**	**% Inhibition**	**Length (cm)**
Model water without any treatment containing mixture of heavy metals and pesticides (+Control)	0.82±0.06	88.77	0.41±0.03	94.38	No growth	100	7.3±0.36
Immobilized P. isolate treated model water containing mixture of heavy metals and pesticides	4.93±0.27	32.47	3.77±0.10	48.36	2.86±0.19	60.82	7.3±0.36
Bioremediation efficacy	56.30%		46.02%		39.18%		-
Each value is given in mean ± SD. Each sample runs in triplicate. Each length (cm) given is average length. Heavy metals and pesticides were used in the concentration of 1x, 2x and 3x. Experimental conditions without toxicants serve as negative control. Bioremediation efficacy calculation: Inhibition % in untreated water - inhibition % in immobilized *P. isolates* treated water at different combination of heavy metals and pesticides concentrations.

**Table 5:** Heavy metal bioremediation by *Pseudomonas fluorescens* AM-1 strain immobilized on alginate beads estimated by AAS.

**Conditions**	**Cr^6+^(ppm)**	**Ni^2+^(ppm)**	**Pb^2+^(ppm)**
Contaminated water with heavy metals treated with immobilized P. isolate	733±7.1	288±5.4	191±4.2
Heavy metal concentrations in water	289±4.7	91±5.10	88±2.6
Percent Bioremediation	60.57%	68.40%	53.93%
Values are ± SD. Percent bioremediation = *(Toxicant concentration before treatment - concentration after treatment x 100) / (concentration before treatment)*
